# Simulation-Assisted Process Design and Experimental Verification of Laterally Confined Oxide Areas Generated with Continuous Electrolytic Free Jet on EN AW-7075 Aluminum Alloy

**DOI:** 10.3390/mi14020293

**Published:** 2023-01-22

**Authors:** Susanne Quitzke, Igor Danilov, André Martin, Roy Morgenstern, Thomas Lampke, Andreas Schubert

**Affiliations:** 1 Professorship Micromanufacturing Technology, Faculty of Mechanical Engineering, Chemnitz University of Technology, 09107 Chemnitz, Germany; 2 Materials and Surface Engineering, Faculty of Mechanical Engineering, Chemnitz University of Technology, 09107 Chemnitz, Germany

**Keywords:** localized mask-less anodization, local functionalization, oxide layer formation, free electrolyte jet, electrodynamic simulation

## Abstract

Local anodization with a free electrolyte jet is a suitable solution for locally confined surface functionalization without additionally required preparation of the parts. However, the geometrical formation of the anodic oxide layer in jet-based anodization is not yet sufficiently understood. In this study, numerical calculations based on physical descriptions are used to describe the lateral and vertical oxide formation on aluminum alloy EN AW-7075. The required electrical resistance and capacitance were determined by immersion-based anodization and implemented into the numerical simulation model to evaluate the electrical conductivity of the porous layer. The simulation results showed an electrical conductivity of 2.6 × 10^−6^ S/m for the porous layer. Subsequently, a model for jet-based anodization was developed and the previous results were implemented to calculate the oxide formation. The simulation results showed decreasing oxide layer thickness at increasing radial distance from the center of the jet, which corresponds to experimental results. The simulation model was validated by varying the current efficiency from 5% to 90%, where similar developments of the anodic oxide layer thickness compared with experimental results were determined at 5%.

## 1. Introduction

Anodic oxidation is an established method for the surface treatment of aluminum alloys. It is an electrochemical process for converting the surface into a porous aluminum oxide layer, which can serve, among other things, as protection against corrosion and wear, as well as an adhesion promoter for paints or a reservoir for lubricants or color pigments [1,2]. In recent years, anodic oxidation of aluminum achieved further widespread applications, e.g., as membranes in filtration and sensor applications, as reservoirs for drugs or catalysts and as templates for the production of nanostructures and nanowires [3,4,5]. According to the state of the art, anodic oxidation is carried out in immersion-based processes that can easily be integrated into industrial process chains and is therefore suitable for industrial scale production.

In immersion-based anodization, the workpiece is usually immersed into an aqueous, acidic solution such as diluted sulfuric acid or oxalic acid and polarized anodically against an insoluble cathode. In the beginning of the process, the reactions of metal dissolution, oxygen evolution, and oxide formation compete at the anode, while hydrogen evolution takes place at the cathode. The overall reaction is described by Equation (1). With increasing thickness, the electrical resistance of the anodic oxide layer increases, which inhibits the reactions of electrochemical metal dissolution and oxygen evolution. In competition with anodic oxide formation, the field-assisted, direct ejection of the aluminum ions into the electrolyte according to Equation (2) and the chemical redissolution of the oxide by the acidic electrolyte according to Equation (3) take place. Both reactions (2) and (3) reduce the efficiency of the electrochemical film formation. According to the oxide growth model of Keller et al. [6], reaction (3) results in pore initiation at weak points in the oxide layer. With increasing pore depth, the current flow is increasingly localized at the bottom of the pore. As a result of the increase in volume due to oxide formation, high compressive stresses arise at the bottom of the pores, since neighboring pore cells hinder each other in their lateral expansion [7]. The layer growth takes place by the plastic flow of newly formed oxide into the pore walls [8,9]. Since the freshly formed porous oxide layer is permanently in contact with the electrolyte, the chemical redissolution according to Equation (3) acts over the entire duration of the process. This is particularly reflected in the continuous widening of the pore channels and leads to a conical pore shape, since the oxide formed firstly in the outer part is exposed to the chemical redissolution for a longer period of time.

(1) $$2\mathrm{Al}+3H_{2}O\to {\mathrm{Al}}_{2}O_{3}+6H^{+}+6e^{-}$$

(2) $$\mathrm{Al}\to {\;\mathrm{Al}}_{\mathrm{ejected}}^{3+}+3e^{-}$$

(3) $${\mathrm{Al}}_{2}O_{3}+6H^{+}\to 2{\mathrm{Al}}^{3+}+3H_{2}O$$

Especially in wear protection applications, it is ecologically and economically reasonable to limit the anodic oxidation to the tribologically stressed functional surfaces. In the immersion-based process, anodic oxidation can only be limited to certain functional surfaces by masking all remaining surfaces. There are high demands on the masking, in particular with regard to tightness, in order to prevent creeping of the current underneath the mask. In the case of small functional surfaces, the effort of applying and removing the masking is high.

Large-area masking can be avoided by applying sealed electrochemical cells to the surface. Electrochemical cells exist in centimeter to micrometer ranges. Hence, small areas can be anodically oxidized using sealed microcapillaries [10]. However, sealed cells can only be used on approximately flat surfaces and are limited in terms of the shape and size of the anodized surface. A commercially used alternative is brush anodizing, which is mainly used to repair anodized surfaces of large components (e.g., directly on aircraft parts) and also enables the local production of wear-resistant layers [11,12]. For localized anodization with lower efforts, adhesive tapes that contain a viscous anodizing medium can be applied to aluminum surfaces [13]. However, the mass transport within the viscous medium is limited and results in lower oxide growth rates and lower oxide thicknesses compared with the conventional immersion-based anodizing process [13].

An alternative process, which does not require masking to realize local anodization and that offers the possibility to address various surface geometries, is anodization with a free electrolyte jet [14]. In jet-based anodization, the shape and the lateral dimension of the anodized area are mainly influenced by the shape of the electrolyte jet. Provided with a constant, circular nozzle diameter, the diameter, the local layer thickness, and the layer thickness distribution are influenced particularly by the process voltage, the working distance, and the electrolyte composition. It was reported that at increasing process voltage, which led to an increasing electric current flow, and at increasing time, the thickness and the diameter of the anodic oxide increased as a result of the higher electrical charge exchange when applying a circular nozzle with 100 µm diameter [14]. Due to the continuous decrease in current density with increasing lateral distance from the nozzle, the layer thickness also decreases continuously. Since there is no sharply defined edge of the oxidized area, its size can be defined, for example, by the area that is enclosed by the first interference maximum on the laser scanning microscope [15]. A reduction in the working distance from 2.5 mm to 0.1 mm along with a voltage decrease from 230 V to 40 V led to a reduction in the diameter of the anodized area to 67% and to an increase in the maximal oxide thickness to 150% [15]. In addition to circular anodic oxide areas, jet-based anodization offers the possibility to create linearly extended and even more complicated oxide areas by controlled movement of the jet over the workpiece surface.

Multiphysics simulations were carried out by Hackert-Oschätzchen et al. [16] for a numerical description of the anodic oxide formation during jet-based anodization. The simulation results showed significant time-dependent effects. In the beginning, the small cross section of the impinging electrolyte jet and its low electrical conductivity compared with the blank metallic workpiece leads to a strong localization of the current flow below the nozzle and laterally confined anodization in the proximity of the jet. The increasing electrical resistance due to the ongoing growth of anodic oxide results in an increasingly wider current density distribution over the surface and hence reduces the localization of the anodic oxide growth [16]. In the simulations, the anodic oxide layer formation was calculated by Faraday’s law according to Equation (4).

(4) $$\frac{h_{\mathrm{Ox}}}{t}=\eta \;\cdot J\;\cdot \;\frac{M_{\mathrm{Ox}}}{\left(1-V_{p}\right)\cdot \;\rho \;\cdot u\;\cdot F}$$

Here, η is the current efficiency, $h_{\mathrm{Ox}}$ is the simulated local anodic oxide layer thickness, t is the anodizing time, J is the current density, $M_{\mathrm{Ox}}$ is the molar mass of the oxide, $V_{P}$ is the volumetric pore fraction in the oxide, ρ is the density of alumina, u is the valence of the aluminum ions, and F is the Faraday constant (96,485 As/mol).

As a further development, the aim of the present study is to create and validate a numerical model of the anodic oxide growth, which offers differentiation between the barrier layer and porous layer in order to predict the local oxide thickness. As a prerequisite, detailed knowledge about the thickness and electrical resistance of the barrier layer as well as the increasing resistance of the growing porous layer is necessary. For this purpose, the values of the polarization resistance ($R_{P}$) and the capacitance of the double-layer ($C_{D}$) during the oxidation of the age-hardenable high-strength aluminum alloy EN AW-7075 were determined in immersion-based anodization. The measured data were used as input parameters for the mathematical description of the anodic oxide layer formation in jet-based anodization. Finally, the model and simulation results were verified with jet-based anodization experiments through comparison with the optically determined, locally confined anodic oxides.

## 2. Materials and Methods

### 2.1. Sample Material and Electrolyte

All the samples were made of EN AW-7075 with a nominal composition (in wt%) of Si ≤ 0.4, Fe ≤ 0.5, Cu 1.2–2.0, Mn ≤ 0.3, Mg 2.1–2.9, Cr 0.18–0.28, Zn 5.1–6.1, and Ti ≤ 0.2. The heat-treatment state was constantly T6, which means the solution was annealed at 470 °C for 1 h with subsequent quenching and artificial aging at 120 °C for 24 h. For comparison with previous results [15], an aqueous solution of 6.1 g/L oxalic acid was used as electrolyte throughout this study, which was supplied as oxalic acid dehydrate (Merck). The electrolyte temperature was maintained at 21.5 °C and the electrolyte reservoir was constantly stirred during anodization. All the used chemicals were of analytical grade. The conductivity, pH, and temperature of the electrolyte were measured with a SevenCompact Duo S213 multi-channel metering device (Mettler Toledo).

### 2.2. Determination of Anodic Oxide and Current Efficiency of Immersion-Based Anodization

For the determination of the oxide mass and related properties, e.g., porosity and current efficiency, a sheet material with the dimensions of 100 × 25 × 1.5 mm^3^ was used. The samples were pretreated by etching in 3 wt% NaOH solution at 60 °C for 3 min and pickling in 1:1 diluted nitric acid at room temperature for 30 s.

Immersion-based anodization was performed at 60 V against a surrounding counter electrode in a beaker with an electrolyte volume of 2 L for 15 min and 30 min. The power supply was a pe1028 (Plating Electronic). The values of the current and voltage were logged internally during the process with an acquisition rate of 1 sample per second. The electrical charge per area was calculated by the integration of the current density transient over the anodizing time. The oxide thickness was measured by optical microscopy on metallographic cross sections, which were prepared by diamond grinding that was accomplished with a finishing process using a silica oxide polishing suspension.

The mass of all samples was determined before and after the dissolution of the anodic oxide in 35 mL/L phosphoric acid + 20 g/L chromium-(VI)-oxide at 60 °C for 4 h with a X1003S balance (Mettler Toledo). Pretests showed that no measurable dissolution of the substrate alloy occurs during the exposition in chromic/phosphoric acid, therefore, the mass difference is equal to the oxide mass. The porosity p of the coatings was estimated by the following formula using the coating mass m, the surface area A, the coating thickness s, and the density of alumina ρ (3.95 g/cm^3^):

(5) p=1−m·A·s·ρ

The current efficiency η was determined by dividing the coating mass *m* by the theoretical mass $m_{\mathrm{th}}$. The theoretical mass $m_{\mathrm{th}}$ was calculated according to Faraday’s law using the electrical charge Q, the molecular mass M of ${\mathrm{Al}}_{2}O_{3}$ (101.96 g/mol), the number of electrons involved in the generation of one ${\mathrm{Al}}_{2}O_{3}$ molecule (*u* = 6), and the Faraday constant F:

(6) $$m_{\mathrm{th}}=\frac{Q\cdot M}{u\cdot F}$$

### 2.3. Electrochemical Impedance Spectroscopy

**Figure 1:**
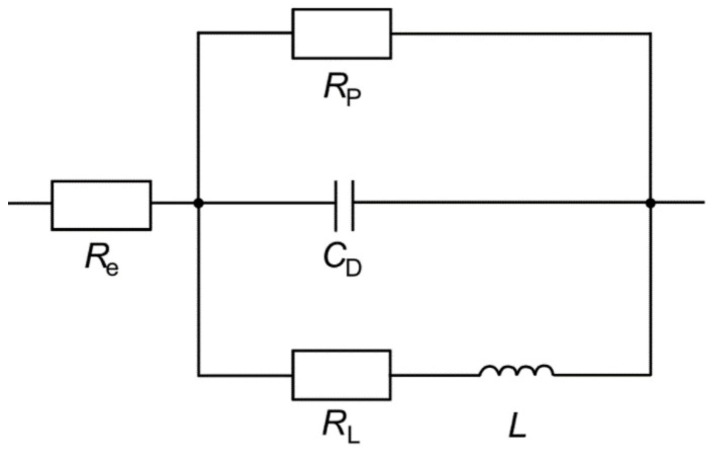
Equivalent circuit used for fitting the EIS results.

Electrochemical impedance spectroscopy (EIS) was applied for the determination of the polarization resistance $R_{P}$ and the double-layer capacitance $C_{D}$. A three-electrode arrangement was used in a beaker with an electrolyte volume of 250 mL at 21.5 °C. EN AW-7075 T6 sheet material was mounted in a sample holder and acted as the working electrode. The active circular surface with a diameter of 10 mm offered an area of approximately 79 mm^2^. A platinum sheet with an area of 15 × 15 mm^2^ was placed in parallel to the working electrode in a distance of approximately 25 mm and acted as the counter electrode. An Ag/AgCl/saturated KCl electrode was used as reference electrode. Throughout the experiment, a constant potential of 60 V was applied to the sample by the electrochemical workstation Zennium X (Zahner). The EIS measurements started after 5 min of polarization in the phase of steady pore growth. For EIS measurement, the constant voltage was overlaid by a sinusoidal measuring voltage with an amplitude of 25 mV. The measurements were performed in a frequency range of 10 kHz–2 Hz with 5 steps per decade and 3 repetitions per frequency. The measurement time of each EIS measurement was approximately 61 s. The results were fitted using the software ZahnerAnalysis provided by the manufacturer of the workstation. The equivalent circuit diagram shown in Figure 1 was derived from Leontiev and Napolskii [17], who also performed EIS measurements during anodizing at constant potential in an oxalic acid solution.

In the equivalent circuit, $R_{e}$ is the resistance of the electrolyte. In serial connection to $R_{e}$ there is another electrical circuit that describes the anodic oxide layer formation through a parallel arrangement of three electrical components: the polarization resistance $R_{P}$, which includes both the resistance of the barrier and the diffusion resistance of the pore channels, the double-layer capacity $C_{D}$, and a serial arrangement of the resistance $R_{L}$ as well as the inductor L. According to the literature, the inductive behavior can be explained by the relaxation of charge-carrying defects [18].

### 2.4. Simulation Model of the Immersion-Based Anodic Oxide Formation

**Figure 2:**
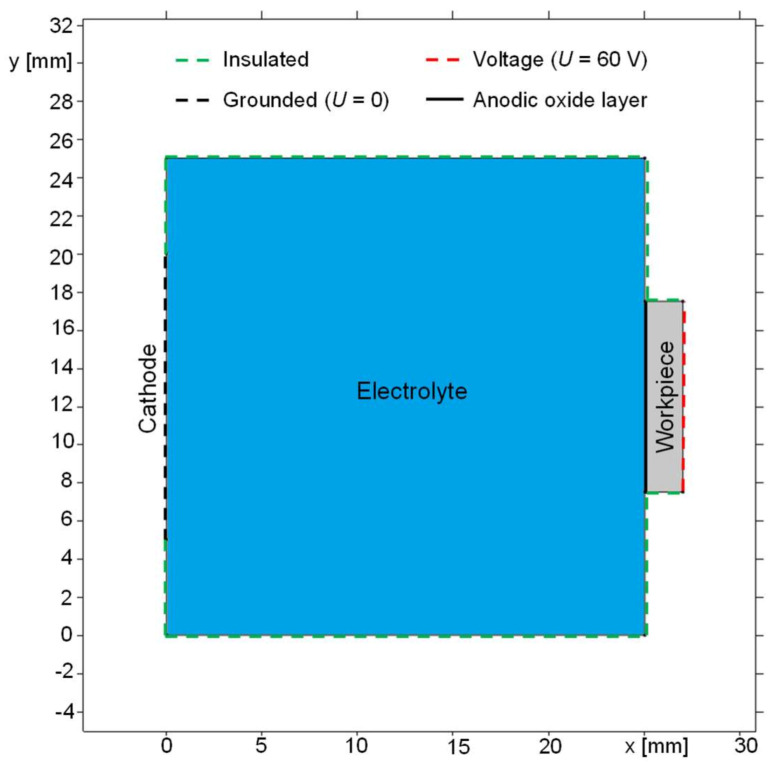
Geometry of immersion-based anodization for parametric simulation.

A model of the immersion-based anodization setup was created in the simulation software COMSOL Multiphysics^®^, which is based on the finite elements method (FEM). Figure 2 presents the geometry and the electrical boundary conditions of the model, where x represents the length and y the height.

The model consists of a workpiece (colored as the grey area) with an anodic oxide layer (bold black line) and connected to an anodic potential of 60 V. The cathode’s surface (black dashed line) with a potential of 0 V and the electric conductivity of AISI 4340 is arranged vertically in parallel to the workpiece surface with a horizontal distance of 25 mm. The cathode’s vertical dimension was set to 15 mm and the anode’s vertical dimension to 15 mm according to the experiment described in Section 2.3. The electrolyte (blue-colored area) realizes the electrical contact between the workpiece and the cathode. The material parameters were the same as in the model described in Section 2.3. The simulation time was set to 30 min in analogy to the experiment.

### 2.5. Simulation Model of the Anodic Oxide Formation in Jet-Based Anodization

**Figure 3:**
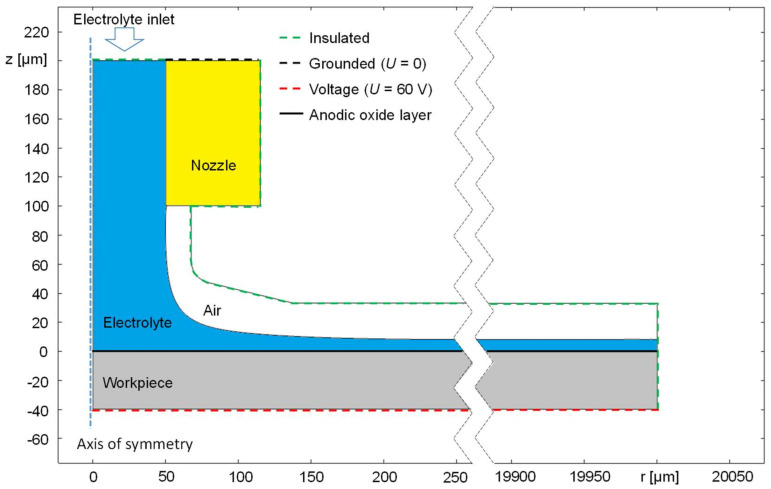
Geometry and electrical boundary conditions of the rotation-symmetric simulation model of jet-based anodization.

In order to describe the potential- and time-dependent anodic oxide formation in jet-based anodization, another model was created according to the geometrical arrangement and the electrical parameters of the respective experimental setup. The distribution of the flow of this model is based on the model described in [19]. The model, which is shown in Figure 3, contains a two-dimensional, rotation-symmetric geometry, where *z* indicates the vertical and *r* the radial dimensions.

The model consists of a steel AISI 4340 nozzle colored in yellow, an electrolyte jet colored in blue, atmospheric air colored in white, and the workpiece anode colored in grey. The nozzle diameter as well as its distance from the anodic workpiece were both set to 100 µm according to the experiments. For the determination of the jet shape, a previous two-phase flow simulation has been conducted considering a radial dimension of r = 300 µm as highlighted by the interruption of the abscissa in Figure 3. For this purpose, an electrolyte flow velocity of 10 mL/min was considered. For means of simplification, the phase distribution at larger radii is assumed to be the same as at r = 300 µm until the maximal radial dimension of the anode’s surface of r = 20,000 µm is reached.

The potential of the nozzle was set to 0 V, while 60 V were applied to the anodic workpiece. The electrical contact between the nozzle and the workpiece is realized via the electrolyte jet. To simulate the oxide layer formation, the upper surface of the anode colored in black (*z* = 0 µm) was defined as a contact impedance layer. The resistance of this layer depends on the thickness of the total oxide that was the sum of the thickness of the barrier layer and the porous layer.

The electrical conductivity of the electrolyte was set to 6.1 S/m according to the measured value of the experiments. For the electrical conductivity of the surrounding air, an arbitrarily small value of 1·10^−12^ S/m was chosen to provide numerical stability. The electrical conductivity of the aluminum anode was set to 23∙10^6^ S/m, and for the nozzle made of stainless steel, 4.032∙10^6^ S/m was chosen according to the value from the material library of the simulation software.

The anodic oxide layer formation in jet-based anodization and thus the conductivity of the oxide layer was unknown and not measurable due to the small lateral dimensions of the local oxides that are not accessible with adequate measuring devices and the complicated distribution of the oxide thickness over the part’s surface. Hence, the electrical conductivities were calculated and evaluated in Section 3.2.1 based on the experimental results of immersion-based anodization. The conductivity of the barrier layer was calculated in Section 3.2.1 based on the experimental data for immersion-based anodization.

The anodic oxide layer formation in this model was described by two differential equations according to (4), which were defined for the barrier layer and the porous layers. $M_{\mathrm{Ox}}$ was 101.96 g/mol, ρ was set to 3.95 g/cm^3^, and u was 6. Equation (4) was solved with an initial thickness of 1 nm for the barrier layer to consider the natural oxide layer and 0 nm for the porous layer.

The calculation of the current density J was carried out according to Equation (7):

(7) $$J=\sigma_{s}\;\cdot \;dV$$

Here, dV is the voltage drop over the formed total oxide layer, which is composed of the barrier layer and the porous layer in series, and $\sigma_{s}$ is a surface electrical conductivity in S/m^2^ that was defined according to Equation (8):

(8) $$\sigma_{s}=\frac{\sigma_{\mathrm{oxide}}}{h_{\mathrm{barrier}}+h_{\mathrm{porous}}}$$

Here, $h_{\mathrm{barrier}}$ and $h_{\mathrm{porous}}$ are the respective thicknesses of the barrier layer and the porous layer and $\sigma_{\mathrm{oxide}}$ is the electrical conductivity of the total oxide layer that depends on the local layer structure, which was defined according to Equation (9):

(9) $$\sigma_{\mathrm{oxide}}=\frac{h_{\mathrm{barrier}}}{h_{\mathrm{barrier}}+h_{\mathrm{porous}}}\;\cdot \;\sigma_{\mathrm{barrier}}+\frac{h_{\mathrm{porous}}}{h_{\mathrm{barrier}}+h_{\mathrm{porous}}}\;\cdot \;\sigma_{\mathrm{porous}}$$

The growth of both the barrier layer and the porous oxide layer was simulated based on the assumption that first the barrier layer grows up, and in the following, the porous layer also begins to grow after full development of the barrier layer according to [20]. The full development of the barrier layer was limited through the maximum of its possible value, which locally depends on the voltage drop over the barrier layer. The thickness of the resulting total oxide layer was calculated as the sum of the time-dependent thicknesses of the barrier and the porous layers.

The simulation time was set to 10 min according to the processing time in the experiments.

### 2.6. Procedure for the Experiments on Jet-Based Anodization

**Figure 4:**
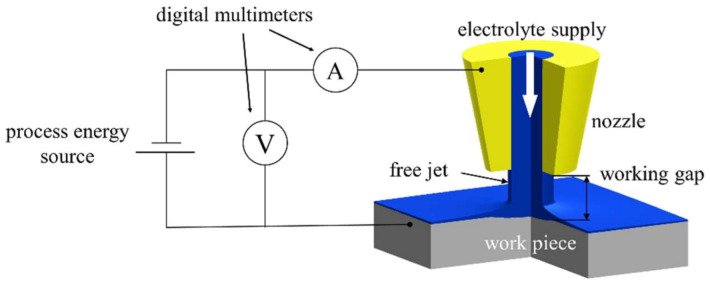
Schematic of the experimental setup for jet-based anodization, modified according to [21].

The jet-based anodization experiments were carried out with a custom Jet-ECM setup of the professorship Micromanufacturing Technology of Chemnitz University of Technology. Figure 4 shows a schematic of the setup.

A detailed description of the setup can be found in [22]. A process energy source N5771 A (Keysight Technologies Inc., Santa Rosa, CA, USA) was used to provide the constant voltage for the experiments. The acquisition of the electric current and the voltage signals was carried out with a customized measuring program based on LabVIEW (National Instruments Corp., Austin, TX, USA) via two digital multimeters 34465 A (Keysight Technologies Inc., USA). Each experiment was carried out three times for statistical evaluation. The process parameters are charted in Table 1.

### 2.7. Measurement of the Local Anodic Oxides after Jet-Based Anodization

The anodic oxides were measured by optical and tactile methods after the experiments. The optical evaluation was carried out with a laser scanning microscope VK-9700 (Keyence) by reflectometric analysis according to [15]. The number of interference rings (i) was counted from the measured laser intensity images using the manufacturer’s analysis software VK Analyzer (Keyence).

The thickness of the anodic oxide ($h_{\mathrm{Ox}}$) was calculated by Bragg’s Equation (10):

(10) $$h_{\mathrm{Ox}}=\frac{i\;\cdot \;\lambda }{2\;\cdot \;\sin\theta \;\cdot \;n}$$

where λ is the wavelength of the laser light, which is 408 nm, *n* is the refractive index of the anodic oxide produced in an oxalic acid electrolyte and exhibits 1.68 according to [23], and the incidence angle of the laser *θ* is 90° in top view analysis arrangement. The radii of the single interference rings were measured from the captured laser intensity images in the cross-sectional direction using the same analysis software as before. The radii were used to analyze the lateral thickness distribution of the anodic oxides over the workpiece surface. Each measurement was carried out three times and the average values as well as the standard deviations were calculated and used for the evaluations.

Tactile measurements were carried out with a roughness and contour measuring device LD 120 (Mahr GmbH, Göttingen, Germany). Constant linear measurements with a length of 5 mm were carried out through the centers of the anodic oxides. The starting point and the ending point were defined as the initial height to compensate the inclination. The evaluation of the profile height as a function of the profile length indicated the anodic oxide layer grown to the outside of the initial surface.

For evaluating the results of the optical measurement, the oxide layer was measured on fractured samples using scanning electron microscopy (SEM). Prior to SEM, bars with a width of about 5 mm were cut out of the samples, whereby each oxidized area was located in the center of the bar length. The bars were bended until fracture using a three-point-bending setup, which was mounted in a mechanical testing machine UPM 1475 (Zwick GmbH & Co. KG, Ulm, Germany). As the samples were in the thermal treatment condition of T6, there was only a little plastic deformation until fracture. Afterwards, the fragments were cleaned in isopropyl alcohol using an ultrasound bath and dried in an oven at 60 °C for at least 5 h. The oxide microstructure was investigated in the vicinity of the fractured surface under a tilt angle of 60° by a field emission scanning electron microscope NEON40EsB (Zeiss AG, Jena, Germany) using the secondary electron (SE) topography contrast detector. For the determination of the exact position of the fracture in relation to the interference rings, laser scanning microscope images of the fragments were captured.

## 3. Results and Discussion

### 3.1. Resistance and Capacitance of Anodic oxides of Immersion-Based Anodization

The time-dependent investigation of $R_{P}$ and $C_{D}$ during immersion-based anodization resulted in the values that are presented in Table 2.

The results indicate that the polarization resistance $R_{P}$ and the double-layer capacitance $C_{D}$ increase slightly with longer anodization time. $R_{P}$ increased from 888 Ω at 1 min to 982 Ω at 30 min. As can be derived from the slight increase in $R_{P}$, the charge carriers must overcome a higher electrical resistance after longer anodization time due to the larger anodic oxide layer thickness to participate in further electrochemical reactions. At the same time, the value of $C_{D}$ almost remains constant considering the measurement deviations. Provided that every single pore with its barrier layer acts as a micro-capacitance, the summed-up total capacitance results from the thickness of the isolator (barrier layer) and the summed-up area of all the pores. This means that the barrier layer thickness and the summed-up area of the pore grounds in the porous anodic oxide layers are approximately similar, when comparing the results after 1 min and after 30 min.

**Figure 5:**
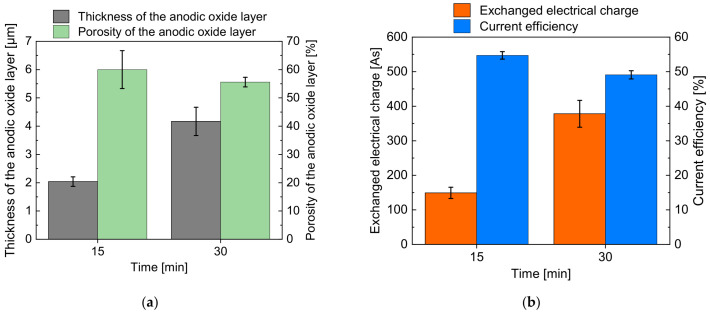
Average values of thickness and porosity of the anodic oxide layer (**a**); exchanged electric charge and current efficiency (**b**) after immersion-based anodization.

In Figure 5a, the realized thickness of the oxide layer (grey column) and its porosity (green column) are presented. The exchanged electric charge (orange column) and current efficiency (blue column) are shown in Figure 5b.

Figure 5a indicates an increase in thickness of the anodic oxidized layer from 2.0 µm after 15 min to 4.2 µm after 30 min. The detected porosity of the oxidized layer was 60% after 15 min and 55.6% after 30 min, which means an almost constant value regarding the measurement uncertainty highlighted by the error bars.

Figure 5b shows an increase in the electric charge from 149 C at 15 min to 378 C at 30 min, which is the cause for the increasing layer thickness at increasing processing time presented in Figure 5a. In contrast, the current efficiency decreased slightly from 54.7% at 15 min to 49.0% at 30 min. This behavior can be explained with the loss of oxide mass due to the progressing re-dissolution of the anodic oxide layer.

### 3.2. Numerical Description of Anodic Oxide Layer Formation

To conduct simulations of the anodic oxide layer formation, the electrical conductivity of the barrier layer and porous layers in the model had to be calculated. The electrical conductivity of the barrier layer was calculated using the experimentally determined values of $R_{P}$ and $C_{D}$ in immersion-based anodization. It is expected that reaction products such as oxygen fill some of the pores during the anodization experiments and thus decrease the electrical conductivity of the porous layer. Hence, the effective electrical conductivity of the porous layer was determined by simulation based on experimental data from immersion-based anodization. The simulation of the jet-based anodization was carried out afterwards using the calculated values of the electrical conductivities.

#### 3.2.1. Electrical Conductivity of the Barrier Layer and Effective Electrical Conductivity of the Porous Layer

The conductivity of the barrier layer was calculated based on Ohm’s law from the data determined in immersion-based anodization using Equation (11):

(11) $$\sigma =\frac{1}{R_{\mathrm{barrier}}}\;\cdot \;\frac{h_{\mathrm{barrier}}}{A}$$

In this equation, $R_{\mathrm{barrier}}$ is resistance of the barrier layer measured in the experiments, A is the surficial area of the sample, and $h_{\mathrm{barrier}}$ is the thickness of the barrier layer. To evaluate the resistance of the barrier layer, the experimental values of $R_{P}$ from the immersion-based process according to Table 2 were used. The time-depending development of the resistance of the oxide layer that was experimentally determined was linearly approximated to the value of 883.93 Ω, which corresponds to the time step of t = 0 min. This constant value was used for the calculation assuming that the initial barrier layer formation is completed within a negligibly short time, when there is no porous layer and the whole resistance only results from the barrier layer.

To evaluate the formed barrier layer thickness, the following Equation (12) was used assuming that the barrier layer has the only contribution to the oxide layer capacitance since the impact of the porous layer can be neglected [17]:

(12) $$h_{\mathrm{barrier}}=\varepsilon \;\cdot \varepsilon_{o}\cdot A/C_{D}$$

Here, ε is the permittivity of aluminium oxide with a value of 8.5 according to [17], $\varepsilon_{o}$ is the vacuum permittivity, A is the surficial area of the sample, and $C_{D}$ is the capacitance of the anodic oxide layer measured in experiments from Table 2. The calculation results is an average value of 50.3 nm for the thickness of the barrier layer and a value of 7.25 × 10^7^ S/m for the electrical conductivity of the barrier layer. The current efficiency η for both oxide layers was set to 0.55 based on the value measured at 15 min in Section 3.1. The pore fraction $V_{P}$ of the porous layer was set to 0.6 based on the value achieved after 15 min. For the barrier layer, $V_{P}$ was set to 0.0 according to [20].

To evaluate the conductivity of the porous layer, a parametric study with the model geometry of the immersion-based anodization (Figure 2) was conducted. The parametric study showed that after 30 min the best agreement with the experimental data was achieved with an electrical conductivity of 2.6 × 10^−6^ S/m, where an average oxide thickness of 4.1 µm was formed, which almost corresponds to the experimental value of 4.2 µm. Hence, the same value of 2.6 × 10^−6^ S/m was used for the simulations of jet-based anodization in Section 3.2.2.

#### 3.2.2. Numerical Description of the Anodic Oxide Formation in Jet-Based Anodization

**Figure 6:**
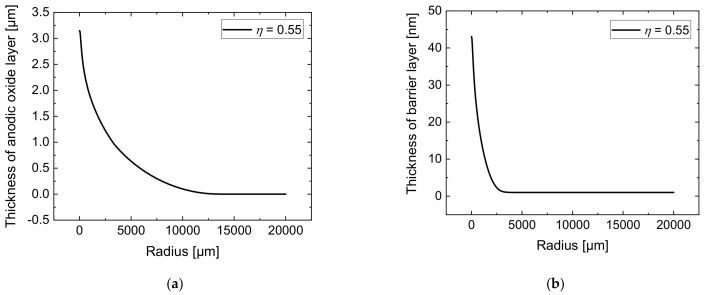
Simulated total anodic oxide thickness on the workpiece surface (**a**) and simulated barrier layer thickness on the anode surface (**b**)—each after 10 min with a current efficiency of η = 0.55 in jet-based anodization.

The simulations of the jet-based anodization process were carried out with a current efficiency of η = 0.55. The results of the simulated thickness distribution of the anodic oxide layer after 10 min are presented in Figure 6a. It can be seen that the anodic oxide exhibits a maximum of 3.15 µm directly under the nozzle’s center and decreases with increasing radial distance. The barrier layer simulated after an anodization time of 10 min can be seen in Figure 6b. Since the formation of the oxide depends on the local voltage drop over the anodic oxide layer, its thickness distribution indicates the respective voltage drop distribution over the anodic surface.

**Figure 7:**
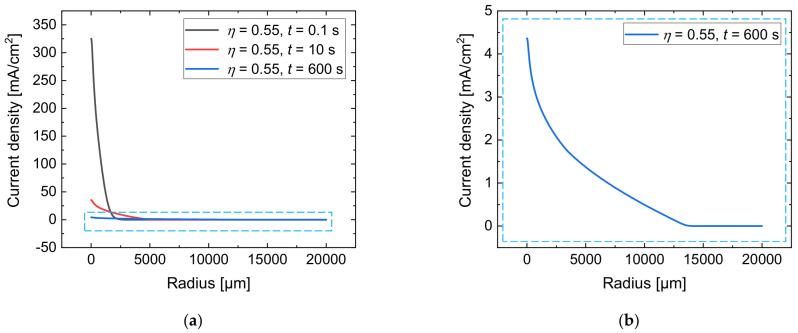
Simulated current density for a current efficiency of η = 0.55 at t = 0.1 s, t = 10 s, and t = 600 s (**a**); detailed view at t = 600 s (**b**).

To describe the radial growth of the anodic oxide layer on the workpiece surface, the current density for a current efficiency of η = 0.55 was calculated based on Equation (7) at various process times. The results are shown in Figure 7a as function of the radius. Figure 7b presents a detailed view of the current density distribution at t = 600 s for a better visibility.

It can be seen that the maximum value of the current density decreases from 325 mA/cm^2^ at t = 0.1 s to 35 mA/cm^2^ at t = 10 s and to 4 mA/cm^2^ after t = 600 s. The growth of the anodic oxide leads to a spreading of the electric current density over the workpiece surface in a radial direction for long distances. Hence, at a radius of 2500 µm, which is 50-fold of the nozzle’s radius, the local current density increases from 0.6 mA/cm^2^ at t = 0.1 s to 9.0 mA/cm^2^ at t = 10 s and to 2.0 mA/cm^2^ after t = 600 s.

**Figure 8:**
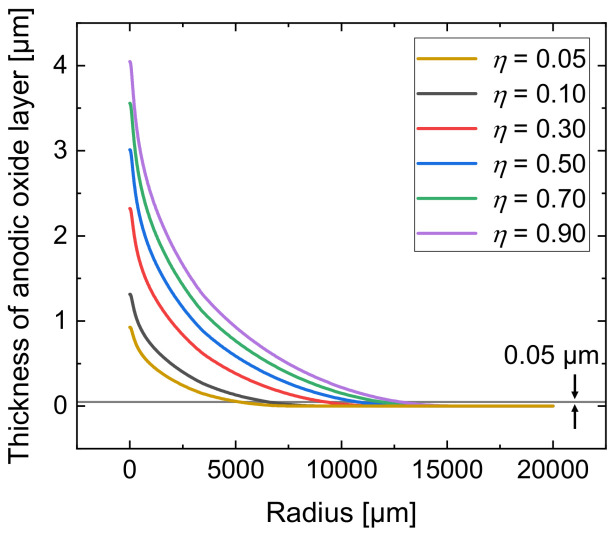
Simulated total anodic oxide thickness after 10 min of the jet-based anodization for different current efficiencies η.

Due to the very small volume of the local oxides, it was hardly possible to determine the mass difference of the samples and the experimental determination of the current efficiency was not possible. Therefore, with the developed simulation model, another parametric study was conducted to determine the influence of the current efficiency η on the local anodic oxide layer thickness. For this purpose, η was varied from 0.05 to 0.90. Figure 8 presents the results.

An increase in anodic oxide layer thickness can be seen with higher current efficiency. The radial trend of the layer thickness is an exponential decrease for all analyzed current efficiencies. At radii larger than 12,500 µm, a layer thickness of only 0.02 µm is achieved, independently of the analyzed current efficiency. Considering significant anodic formation that exceeds the barrier layer thickness and can therefore be defined with a minimal thickness of 0.05 µm, the lateral dimension depends on η as highlighted by the horizontal line in Figure 8. While a current efficiency of η = 0.90 results in a radial expansion of approximately 14,150 µm, only 6470 µm are achieved with η = 0.05. Thus, the lateral expansion of the anodic oxide layer is significantly reduced at lower current efficiency.

### 3.3. Measured Thickness of the Anodic Oxide Layer after Jet-Based Anodization

**Figure 9:**
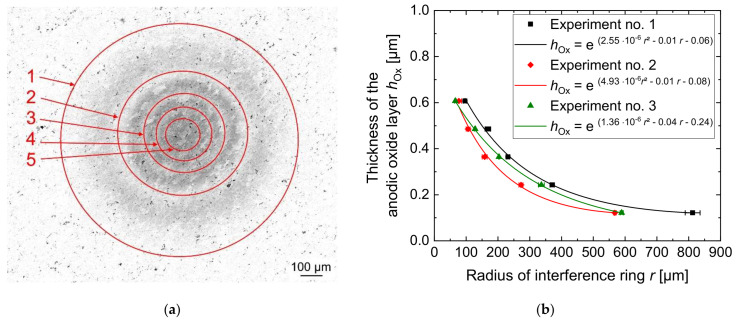
Optical evaluation of the local anodic oxides by laser intensity images with marked interference rings (**a**) and thickness characteristic of the anodic oxide as a function of the radius of the interference rings (**b**) after jet-based anodization. Linear trends indicate exponential interpolations considering radial distances within µm values.

Five interference rings were detected by optical analysis of the oxidized area in all experiments as can be seen in Figure 9a. Additional rings in the center of the oxidized area could not be separated because they were too close to each other and, therefore, their distances were below the resolution of the microscope.

The measured radii of the interference rings are shown in Figure 9b. An exponential fit was used to illustrate the radial decrease in the anodic oxide layer thickness.

Figure 9b shows that the largest anodic oxide thickness was achieved in the middle of the anodized area with a value of 0.61 µm directly below the center of the nozzle. At this position, the current density had the highest value according to the results shown in Figure 8. Therefore, the highest electric charge was exchanged at this position. From the center of the anodic oxide, the thickness decreases to a value of 0.12 µm at approximately *r* = 600 µm. This correlates with the calculated distribution of the current density over the aluminum surface in Figure 7, which means that the anodic oxide growth rate is lower in the outer areas with larger distance from the center of the electrolyte jet.

Since the distribution of the anodic oxide layer thickness on the aluminum workpiece surface agrees with the distribution of the current density calculated from the numerical model, the model for the jet-based anodization was verified. However, the optically measured height of the anodic oxide layer is significantly smaller than the calculated height in Figure 6. It is therefore assumed that a significant amount of the exchanged electric charge was consumed by the formation of gaseous oxygen, which was not considered in the simulation model.

**Figure 10:**
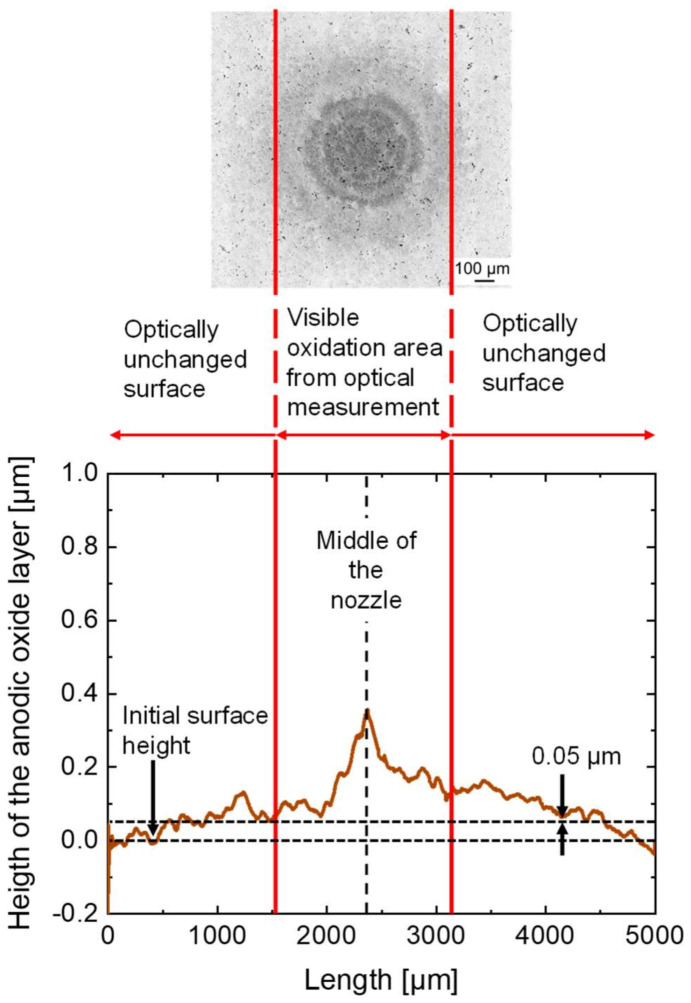
Result of the tactile measurement of the oxide area after the jet-based anodization—experiment no. 3.

An exemplary result of the tactile measurements of the anodic oxides is presented by the brown-colored line in Figure 10 showing a linear topography smoothened with 1000 points. A surface line, which is positioned in parallel to the abscissa, was plotted into the diagram to mark the initial surface height. At a height of 0.05 µm, a second line was added to illustrate significant anodic oxide formation exceeding the barrier layer thickness.

The red-colored vertical lines mark the area, which was determined as the outer dimension by optical measurement based on the detected interference lines. In the tactile measurement, the profile line shows an increasing height of the anodic oxide layer to a maximum of 0.35 µm. The maximum was reached in the middle below the center of the nozzle and has a radially decreasing trend.

The optically unchanged surfaces at radial distances below 1500 µm and above 3200 µm mark the transition areas between the anodized surface and the surrounding initial surface. In these areas, no further interference rings were visible in the optical measurements, since the total anodic oxide layer thickness is too thin for interference. However, due to the wetting with the electrolyte, anodic passive layer formation and oxide dissolution still take place.

**Figure 11:**
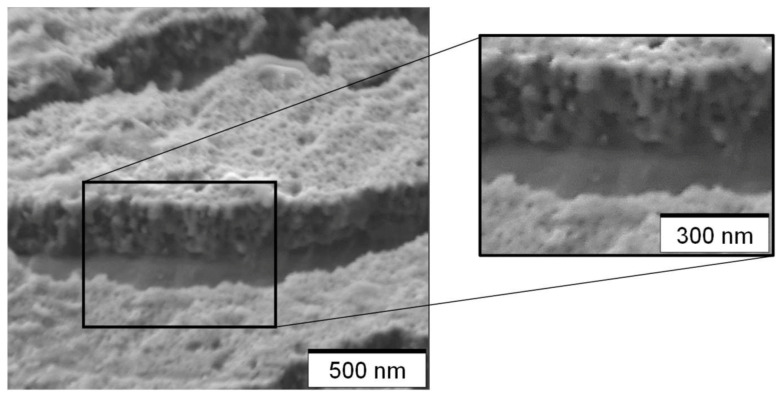
Secondary electron image of the fractured oxide in close proximity to the fracture edge under a tilt angle of approximately 60°.

A SEM image of the fractured anodic oxide in close proximity to the fracture edge is shown in Figure 11.

Considering the tilt angle of 60° referred to the horizontal position, which is reduced by about 5 to 10° due to necking, the oxide thickness is between 275 nm and 295 nm. This is the area with the largest thickness along the fractured edge. Subsequent LSM imaging and light microscopy showed that the fracture does not go exactly through the middle of the anodic oxide. In more detail, light microscopy identified the second order of the interference color cyan in close proximity to the fracture edge, which correlates with a thickness of approximately 300 nm. Considering the inaccuracies being related to positioning and the greatest observed necking angle of 10° being close to the fracture edge, this is in good agreement with the SEM measurement. Hence, the optical thickness measurement was proved to be reliable.

Furthermore, Figure 11 shows a non-conformal pore structure which varies significantly from a highly self-ordered pore structure. A comparable structure was also found for anodizing with the same voltage and electrolyte in immersion-based [24] and in jet-based anodization [15].

### 3.4. Validation of the Numerical Description of the Local Anodic Oxide Layer Formation

**Figure 12:**
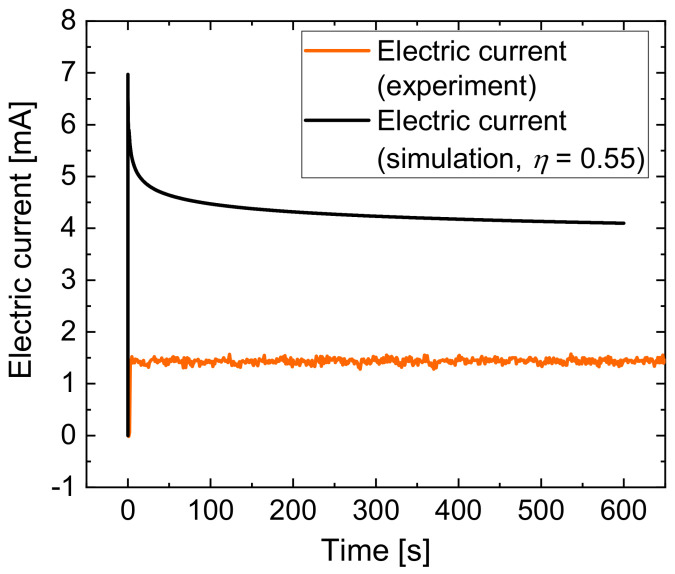
Comparison of the experimentally determined and the calculated electric current during jet-based anodization.

To validate the simulation model, the electric current developments over the process time of the simulation and the experiment for jet-based anodization were compared. Figure 12 shows the experimentally determined (orange line) and the calculated (black line) electric current characteristics. The simulated current achieved a maximum of 6.97 mA in the beginning of the process and a declining decrease to 4.1 mA at the end of the simulation, which was 600 s.

The comparison showed that the current values from the simulation results are approximately three times higher than the experimental values. This explains why the thickness of the anodic oxide in the simulation was higher than in the experiments. This comparison also shows that the total resistance of the process in the simulation is lower than in the experiment. A possible explanation is the fact that the simulation did not take into account the formation of oxygen and the development of other reaction products, which significantly influence the local conductivity of the electrolyte.

The current efficiency of jet-based anodization was calculated based on the tactile measurement results. For this purpose, the measured heights and the radii were used to calculate the volume of the anodic oxide grown outwards, which was considered a conical shape. The comparison between the optically measured thickness of the profile in Figure 9b and the tactilely measured profile in Figure 10 shows that the anodic oxide layer thickness at a defined distance from the center is about twice of the profile height. It follows that half of the anodic oxide layer has grown outward and half inward of the sample. For additional consideration of the anodic oxide grown in the inward direction, twice the conically shaped volume was calculated and defined as the experimental volume. The theoretical volume was calculated via Faraday’s law from the measured electric charge exchange under consideration of the density and porosity of the anodic oxide. The division of the experimental volume divided by the theoretical volume resulted in a current efficiency of 0.05.

**Figure 13:**
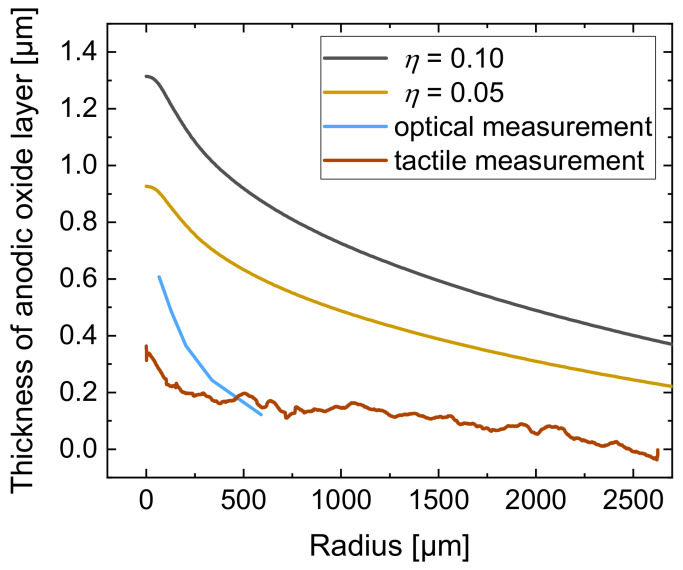
Comparison of the calculated anodic oxide thickness at η = 0.10 (black line) and η = 0.05 (yellow line) with the anodic oxide thickness from optical (blue line) and tactile (brown line) measurements as a function of the radius.

Hence, it can be stated that the current efficiency of 0.55 for jet-based anodization was over estimated. Because of this, the experimental data of the realized oxide thickness were compared with simulation results for lower values of current efficiency. The comparison of the calculated anodic oxide thicknesses at η = 0.10 (black line) and η = 0.05 (yellow line) with the optically (blue line) and tactilely (brown line) determined anodic oxide thicknesses is shown in Figure 13. For the comparative evaluation only, the segment −200 ≤ r ≤ 2700 µm is shown here.

Figure 13 shows that the optically and tactilely measured anodic oxide thicknesses are smaller than the calculated thicknesses at η = 0.10, where the maximal anodic oxide height was 1.2 µm. A reduction in η to 0.05 shows a smaller maximal height with a thickness of 0.93 µm.

The optically measured maximal anodic oxide thickness was 0.61 µm and the tactilely measured anodic oxide layer thickness was 0.35 µm, which significantly differs from the calculated thicknesses. The overestimation for the maximum thickness of the oxide in the simulation can also be explained with the higher electrical current in the developed model.

However, the basic characteristic of the anodic oxide with decreasing thickness at increasing radial distance shows similar tendencies when comparing the experiment with the simulation results. In order to predict the real anodic oxide growth more precisely, a reduction in the current efficiency in the simulation model was identified to be useful to consider secondary chemical reactions.

The results show that a lower current efficiency was achieved in jet-based anodization than in immersion-based anodization. Thus, the proportionate consumption of electric charge for the chemical redissolution of the anodic oxide in the acidic electrolyte, for both electrochemical metal dissolution of the aluminum alloy and for gaseous oxygen evolution, are significantly higher in jet-based anodization compared with immersion-based anodization.

## 4. Summary and Conclusions

The main conclusions are summarized in the following points:

(1) The immersion-based anodization of the aluminum alloy EN AW-7075 shows that the polarization resistance and the capacitance of the double layer hardly changed with longer lasting anodization times. Longer processing time led to a larger anodic oxide thickness, while the porosity remained about constant.
(2) A simulation model considering the development of the barrier layer and the subsequent porous layer formation was developed to describe the anodization process. An electrical conductivity of 2.6∙10^−6^ S/m of the porous layer was determined when anodizing with oxalic acid. The maximum height in the center and the radial decrease in the anodic oxide thickness in the jet-based anodization is caused by the characteristic distribution of the current density over the workpiece surface.
(3) Jet-based anodization with constant DC voltage is a useful technique to create locally confined anodic oxides without masking. Anodic oxides with a maximal layer thickness of approximately 0.61 µm and a diameter of approximately 1700 µm were realized after 10 min. The exact transition between the local oxide and the surrounding surface was difficult to be clearly identified. Further measurements with alternative setups offering higher resolution should be considered. Pulsed voltages could be helpful to use the charging time of the double layer capacity in order to achieve a stronger localization and more sharp-contoured boundaries.
(4) Due to simplifications in the simulation model, the electric current differed from the experiment because the formation of gaseous oxygen, the electrochemical metal dissolution, and the chemical redissolution of anodic oxide were not considered. A parametric study of the current efficiency indicated only low values for jet-based anodization. Significantly lower values compared with immersion-based anodization can be explained by higher proportional consumption of the exchanged electric charge for secondary reactions such as gaseous oxygen production due to the characteristic current density distribution in jet-based anodization.

## Figures and Tables

**Table 1:** Process parameters of jet-based anodization experiments.

Parameter	Value
Conductivity of the electrolyte	6.1 S/m (at 21.5 °C)
pH value	0.8 (at 21.5 °C)
Electrolyte flow velocity	10 mL/min
Processing time	10 min
Working gap	100 µm
Diameter of the electrolyte nozzle	100 µm
DC voltage	60 V

**Table 2:** Experimentally investigated values of $R_{P}$ and $C_{D}$ for immersion-based anodization.

Process Time [min]	*R*_P_ [Ω]	*C*_D_ [nF]
1	888 ± 201	115 ± 8
5	899 ± 192	116 ± 8
10	913 ± 181	117 ± 9
30	982 ± 150	120 ± 13

## Data Availability

Not applicable.
